# Intimate Partner Violence–Related Homicides of Hispanic and Latino Persons — National Violent Death Reporting System, United States, 2003–2021

**DOI:** 10.15585/mmwr.ss7309a1

**Published:** 2024-12-12

**Authors:** Sarah Treves-Kagan, Yanet Ruvalcaba, Daniel T. Corry, Colleen M. Ray, Vi D. Le, Rosalyn D. Lee, Carlos Siordia, Melissa C. Mercado, Lianne Fuino Estefan, Tatiana M. Vera, Megan C. Kearns, Laura M. Mercer Kollar, Delight E. Satter, Ana Penman-Aguilar, José T. Montero

**Affiliations:** ^1^Division of Violence Prevention, National Center for Injury Prevention and Control, CDC, Atlanta, Georgia; ^2^Injury Prevention Research Center, The University of Iowa, Iowa City, Iowa; ^3^Columbia University, Teachers College, Department of Counseling and Clinical Psychology, New York, New York; ^4^Hispanic Association of Colleges and Universities National Internship Program, San Antonio, Texas; ^5^Division of Overdose Prevention, National Center for Injury Prevention and Control, CDC, Atlanta, Georgia; ^6^National Center for State, Tribal, Local, and Territorial Public Health Infrastructure and Workforce, CDC, Atlanta, Georgia; ^7^Elder and tribal member of the Confederated Tribes of Grand Ronde; ^8^Office of Health Equity, CDC, Atlanta, Georgia

## Abstract

**Problem/Condition:**

In 2022, homicide was the second leading cause of death for Hispanic and Latino persons aged 15–24 years in the United States, the third leading cause of death for those aged 25–34 years, and the fourth leading cause of death for those aged 1–14 years. The majority of homicides of females, including among Hispanic and Latino persons, occur in the context of intimate partner violence (IPV). This report summarizes data from CDC’s National Violent Death Reporting System (NVDRS) on IPV-related homicides of Hispanic and Latino persons in the United States.

**Period Covered:**

2003–2021.

**Description of System:**

NVDRS collects data regarding violent deaths in the United States and links three sources: death certificates, coroner or medical examiner reports, and law enforcement reports. IPV-related homicides include both intimate partner homicides (IPHs) by current or former partners and homicides of corollary victims (e.g., children, family members, and new partners). Findings describe victim and suspect sex, age group, and race and ethnicity; method of injury; type of location where the homicide occurred; precipitating circumstances (i.e., events that contributed to the homicide); and other selected characteristics. Deaths related to each other (e.g., an ex-partner kills the former partner and their new partner) are linked into a single incident. State participation in NVDRS has expanded over time, and the number of states participating has varied by year; data from all available years (2003–2021) and U.S. jurisdictions (49 states, Puerto Rico, and the District of Columbia) were used for this report. Of the 49 states that collect data, all except California and Texas collect data statewide; Puerto Rico and District of Columbia data are jurisdiction wide. Florida was excluded because the data did not meet the completeness threshold for circumstances.

**Results:**

NVDRS collected data on 24,581 homicides of Hispanic and Latino persons, and data from all available years (2003–2021) and U.S. jurisdictions (49 states, Puerto Rico, and the District of Columbia) were examined. Among homicides with known circumstances (n = 17,737), a total of 2,444 were classified as IPV-related (13.8%). Nearly half of female homicides (n = 1,453; 48.2%) and 6.7% (n = 991) of male homicides were IPV-related; however, among all Hispanic and Latino homicides, most victims were male (n = 20,627; 83.9%). Among the 2,319 IPV-related homicides with known suspects, 85% (n = 1,205) of suspects were current or former partners for female victims, compared with 26.2% (n = 236) for male Hispanic and Latino victims. Approximately one fifth (71 of 359 [19.8%]) of female IPV-related homicide victims of childbearing age with known pregnancy status were pregnant or ≤1 year postpartum. Approximately 5% of IPV-related homicide victims were identified as Black Hispanic or Latino persons (males: n = 67; 6.8%; females: n = 64; 4.4%). A firearm was used in the majority of Hispanic and Latino IPV-related homicides (males: n = 676; 68.2%; females: n = 766; 52.7%).

**Interpretation:**

This report provides a detailed summary of NVDRS data on IPV-related homicides of Hispanic and Latino persons in the United States during 2003–2021. This report found heterogeneity of characteristics and circumstances of Hispanic and Latino IPV-related homicides. Whereas most Hispanic and Latino homicide victims were male, nearly 60% of Hispanic and Latino IPHs and IPV-related homicide victims were female. Additional research is needed to better understand the relation between IPHs and IPV-related homicides and race (distinct from ethnicity) and pregnancy.

**Public Health Action:**

NVDRS provides critical and ongoing data on IPV-related homicides of Hispanic and Latino persons in the United States that can be used to identify existing strategies and develop new early intervention strategies to prevent IPV and the escalation of IPV to IPH. Strategies that have demonstrated promise in reducing rates of IPH include expanded availability of low-income housing units; sanctuary policies that outline the relation between immigration enforcement and law officers; state laws prohibiting firearm access to those subject to domestic violence restraining orders; improvement of community relations with police to implement risk-based interventions; and comprehensive social, economic, medical, and legal safety nets to create pathways out of abusive relationships, including for pregnant women. Community, local, state, and Federal leaders can combine data on IPV-related deaths and the best available evidence-based programming and policy to create community-engaged solutions that reflect the experience of their Hispanic and Latino communities, including historical and societal factors that increase risk for violence.

## Introduction

In the United States in 2022, homicide was the second leading cause of death for Hispanic and Latino persons aged 15–24 years, the third leading cause of death for those aged 25–34 years, and the fourth leading cause of death for those aged 1–14 years (*1*). Intimate partner violence (IPV) is defined as physical or sexual violence, stalking, or psychological harm by a current or former intimate partner or spouse (*2*). Intimate partner homicide (IPH) is a lethal form of IPV and accounts for a considerable proportion of homicides. In 2020, for all race and ethnicity groups, when the relationship of victims to homicide suspects was known, the suspects were current or former intimate partners of female (50.0%) and male victims (7.9%) (*3*). IPV-related homicides include homicides by current or former partners (i.e., IPHs) and homicides of corollary victims (e.g., children, family members, new partners, friends and acquaintances of intimate partners, law enforcement officers, and strangers who might have been present at the time of the incident). Using this more expansive definition allows for better understanding of the full extent of lives lost to IPV-related homicide. An analysis of National Violent Death Reporting System (NVDRS) data from 2006–2015 across 16 states found that approximately 290,000 potential years of life were lost because of IPV-related homicides (*4*).

Multiple analyses of NVDRS data also have identified disparities in IPV-related homicide victimization by sex as well as race and ethnicity (*4*–*6*). Although men account for approximately three fourths of firearm-related homicide victims in the United States, women account for approximately three fourths of firearm-related IPH victims (*7*). An analysis of NVDRS data collected during 2003–2014 in 18 states found that 61% of homicides (using any weapon and method type) of female Hispanic and Latino persons were IPV-related (*5*). A 2021 study found that Hispanic and Latino persons lost approximately 33,500 potential years of life from IPV-related homicides (*4*); both Hispanic and Latino IPHs and corollary victims were younger than their White counterparts, leading to disparities in potential years of life lost (*4*). Because the Hispanic and Latino population is now the largest ethnic minority group in the United States, and majority population in certain areas, additional research on IPV-related homicides among this group is needed to improve prevention efforts (*8*).

### Hispanic and Latino Populations in the United States

The Hispanic and Latino community is diverse with important and increasing economic, political, and cultural influence throughout the United States (*9*,*10*). During 2010–2020, approximately half (51.1%) of the population growth in the United States was attributable to Hispanic and Latino persons (*10*). After the 2020 census, the U.S. Census Bureau estimated there were 62.1 million Hispanic and Latino persons in the United States, accounting for 18.7% of the country’s population (*10*), although this is likely an undercount (*11*). Hispanic and Latino communities also are racially diverse. The American Community Survey’s 5-year estimate during 2017–2021 found that 47.3% of Hispanic and Latino persons identified as White, 28.2% as another race, and 20.7% as multiracial. Approximately 2.0% of respondents selected Black or African American (Black) alone, 1.3% selected single-race American Indian or Alaska Native (AI/AN), and <1.0% selected Native Hawaiian or other Pacific Islander or Asian only (*12*). Many data collection systems and data users’ analytic approaches do not include the full diversity of Hispanic and Latino persons, specifically indigenous populations and descendants of enslaved Africans in the United States, Latin America, and the Caribbean (*13*–*15*).

Although the term “Hispanic and Latino” is used in this report, the social constructs of ethnicity and the terminology used to describe it can vary by context and evolve (e.g., “Latinx” or “Latiné”). In the United States, the term “Hispanic” is used to refer to persons from Spanish-speaking countries and places (e.g., Spain, Mexico, and Puerto Rico). The term “Latino” includes persons from Latin American countries, including those who are not Spanish speaking (e.g., Brazil), and certain Caribbean countries. Because the term “Latino” is considered by certain persons as a masculine demonym that erases “the existence of other gendered possibilities” (*16*), the term “Latinx,” emerged and is mostly used in the United States, replacing the “a” and “o” signifiers of masculine and feminine forms, rejecting gender binaries. “Latiné” is a more popular term in Latin America that also provides a gender-neutral alternative and is more “phonologically attuned and fluid” in Spanish (*16*). Mixed support for each term exists; more in-depth discussions of the nuances of each term and the debate regarding which term is preferred are published across the research literature (*16*,*17*).

The current and historical social and structural conditions in which Hispanic and Latino persons live, work, and learn can influence their risk for multiple forms of violence. Research on risk and protective factors for IPH continues to grow (*18*), expanding what is known among different racial and ethnic communities. Studies suggest that Hispanic and Latino communities can disproportionately experience risks for IPV because of structural marginalization, interpersonal discrimination, and xenophobia (*19*–*23*). Poverty and economic hardships also can increase the risk for IPV (*24*–*27*), and research has consistently documented the relation between IPV and housing instability and homelessness for women and children (*24*,*25*,*28*,*29*). Certain Hispanic and Latino communities in the United States experience economic marginalization (*30*–*32*). For example, in 2022, Hispanic and Latino women earned 85% as much as Hispanic and Latino men and 65% as much as non-Hispanic White men (*33*,*34*). Having multiple, marginalized identities also can magnify risk for violence, and trauma exposures can persist across generations (*35*–*38*). Hispanic and Latino persons, particularly those with indigenous and African ancestry, have experienced profound effects of past and current systemic marginalization and intergenerational trauma resulting from massacres; enslavement; sexual abuse; loss of land, language, and culture; systems marginalization; and forced acculturation (*36*–*38*). These effects are associated with IPV through multiple mechanisms, including higher household stressors, lower access to services, and vulnerability to revictimization (*21*,*23*,*39*). However, many Hispanic and Latino communities in the United States also hold a strong sense of cultural heritage, value community, place substantial importance on family, and demonstrate considerable resilience, which are all important protective factors for multiple forms of violence (*39*,*40*).

Responding to and preventing IPV-related homicides of Hispanic and Latino persons requires characterizing victim and suspect demographics, the relations between victims and suspects, and the contexts in which the homicides occur. This report provides an overview of IPV-related homicides of Hispanic and Latino persons in the United States using a state-based surveillance system. Specifically, this report presents data from NVDRS to help Federal, tribal, state, and local governments; public health and health services sectors; the criminal justice system; and victim services to better understand homicide circumstances and inform prevention efforts.

## Methods

NVDRS is an active, state-based surveillance system that collects information from death certificates, coroner or medical examiner reports, and law enforcement reports on the characteristics and circumstances of violent deaths, including homicides (*41*). NVDRS combines information for each death and links related deaths (e.g., multiple homicides and homicide followed by suicide occurring within 24 hours) into a single incident. Trained data abstractors code up to 600 variables using standardized guidance from CDC and write detailed narratives about each incident based on coroner or medical examiner reports and law enforcement reports. Data abstractors select from a list of potential circumstances and are required to code all circumstances that are known to relate to each incident; therefore, circumstances are not mutually exclusive. If either the coroner or medical examiner report or law enforcement report indicates the presence of a circumstance, then the abstractor endorses the circumstance. Abstractors enter this information into an encrypted, web-based system. If circumstances are unknown (e.g., a body was found in the woods with no other details reported), the death was excluded from the denominator for circumstance values.

State participation in NVDRS has expanded over time, and the number of states participating has varied by year (*42*,*43*) (Figure) (Supplementary Table 1, https://stacks.cdc.gov/view/cdc/169628). Data from all available years (2003–2021) and U.S. states, jurisdictions, and territories (49 states, Puerto Rico, and the District of Columbia) were used for this analysis. Florida was excluded because the data did not meet the completeness threshold for circumstances. Because Puerto Rico is a unique social, economic, and political location for Hispanic and Latino populations in the United States, a supplemental analysis also was conducted using only data from Puerto Rico. NVDRS data are updated annually and are available to the public through the Web-based Injury Statistics Query and Reporting System (https://www.cdc.gov/injury/wisqars/nvdrs.html). For this report, case-level NVDRS data were used through the NVDRS Restricted Access Database (https://www.cdc.gov/nvdrs/about/nvdrs-data-access.html).

**FIGURE:**
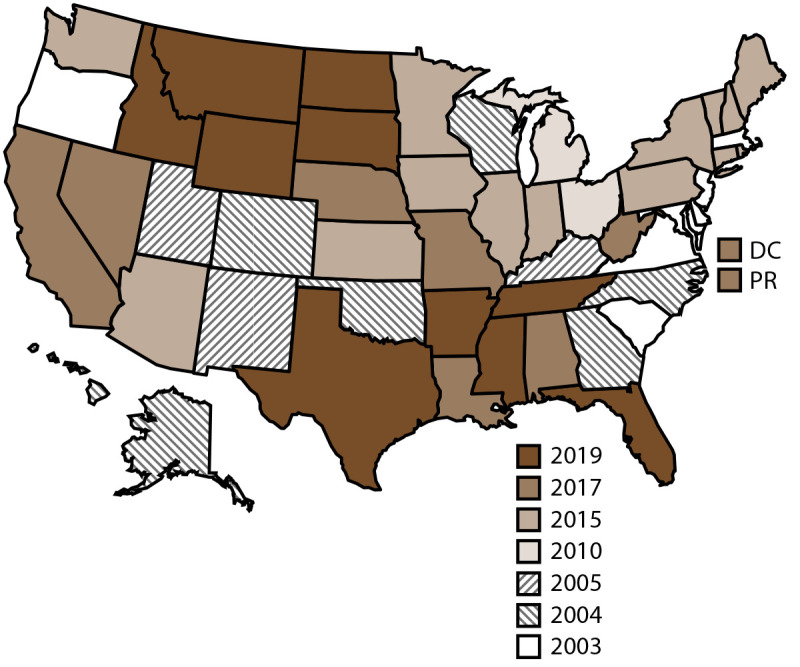
States and jurisdictions participating in the National Violent Death Reporting System, by year of initial data collection* — United States and Puerto Rico, 2003–2021 **Abbreviations:** DC = District of Columbia; NVDRS = National Violent Death Reporting System; PR = Puerto Rico. * Map of United States indicates the year in which the state or jurisdiction began collecting data in NVDRS. Beginning in 2019, all 50 U.S. states, the District of Columbia, and Puerto Rico were participating in the system. California began collecting data for a subset of violent deaths in 2005 but ended data collection in 2009; however, in 2017, California resumed collecting data for a subset of violent deaths and expanded coverage in subsequent years. In 2021, California collected data for violent deaths in 31 counties (Amador, Butte, Colusa, Fresno, Glenn, Humboldt, Imperial, Kings, Lake, Lassen, Los Angeles, Mendocino, Merced, Modoc, Mono, Orange, Placer, Sacramento, San Benito, San Diego, San Francisco, San Luis Obispo, San Mateo, Santa Cruz, Shasta, Siskiyou, Solano, Sonoma, Tehama, Ventura, and Yolo) representing 62% of the state’s population. Michigan collected data for a subset of violent deaths during 2010–2013 and expanded to collecting statewide data beginning in 2014. In 2016, Illinois, Pennsylvania, and Washington began collecting data on violent deaths in a subset of counties that represented at least 80% of all violent deaths in their state or in counties. Washington began collecting statewide data for all violent deaths beginning in 2018, and Illinois and Pennsylvania began collecting statewide data beginning in 2020. In 2019, Texas began collecting data for a subset of violent deaths and expanded coverage in subsequent years. In 2021, Texas collected data for violent deaths that occurred in 13 counties (Bell, Bexar, Collin, Dallas, Denton, El Paso, Fort Bend, Harris, Montgomery, Nueces, Tarrant, Travis, and Williamson) representing approximately 63% of the state’s population.

### Definitions and Variables Analyzed

Data reported include homicide victim details and circumstances, and when available, suspect details; variable definitions followed standardized NVDRS coding guidance (*42*). NVDRS defines homicide as a death resulting from the use of physical force or power, threatened or actual, against another person, group, or community when a preponderance of evidence indicates that the use of force was intentional. Homicides were classified by the *International Classification of Diseases*, Tenth Revision cause-of-death codes X85–X99, Y00–Y09, Y87.1, and U01–U02 (*44*). In this report, victims and suspects with Mexican, Puerto Rican, Cuban, Central or South American, or other Spanish culture or origin are considered Hispanic or Latino persons, regardless of race. Precipitating circumstances are defined as the events that contributed to the infliction of a fatal injury and are reported on the basis of the content of coroner or medical examiner and law enforcement investigative reports.

IPV-related deaths were defined as those involving homicides committed by intimate partners (i.e., victim was an intimate partner or spouse [current, former, or unspecified] of the suspect), corollary victims of IPH (i.e., other deaths associated with IPV, including homicides of victims who were not the intimate partner, such as family, friends, others who intervened in IPV, first responders, and bystanders), or homicides precipitated by jealousy or distress over an intimate partner’s relationship or suspected relationship with another person.

Variables examined include characteristics of victims and suspected perpetrators (suspects), incidents (e.g., when and where the incident occurred), weapons that inflicted fatal injuries, and circumstances that preceded and directly contributed to the death (e.g., IPV). Data on these precipitating circumstances often originate from investigators’ interviews with informants who knew the victim, witnessed the incident, or both. No personally identifying information is entered into the NVDRS web-based system.

### Analysis

Analyses were conducted for all homicides of Hispanic and Latino victims and the subset of IPV-related homicides among Hispanic and Latino persons (e.g., national dataset); this approach builds from previous NVDRS analyses for other populations (*5*,*6*). Descriptive analyses were conducted for sociodemographic characteristics of victims and suspects (i.e., sex, age, race and ethnicity, education, metropolitan status, and pregnancy status), mechanisms used to inflict fatal injuries (e.g., firearms), and incident characteristics (i.e., location of injury and victim’s relationship to the suspect). Rural-urban commuting area codes were used to classify geographic areas into metropolitan and nonmetropolitan categories. Sexual orientation and transgender identity were not included in analyses because of challenges collecting these data (*45*). Categories of precipitating circumstances included interpersonal problems, interpersonal conflict (e.g., IPV or family relationship problem), life stressors (e.g., crisis during previous or upcoming 2 weeks), crime and criminal activity (e.g., drug involvement), and other events of the homicide that were relevant to the death (e.g., victim used a weapon).

All descriptive analyses were conducted and independently confirmed using R (version 4.0.3; R Foundation) and Stata (version 18.0; StataCorp). Detailed descriptions of IPV-related homicide victims, homicide circumstances, and suspect characteristics are presented (Tables 1, 2, 3, and 4). Details for all Hispanic and Latino homicide victims, homicide circumstances, and suspect characteristics also are presented (Supplementary Tables 2, 3, and 4, https://stacks.cdc.gov/view/cdc/169628), as are characteristics of IPV-related homicides, circumstances, and suspects in Puerto Rico (Supplementary Table 5, https://stacks.cdc.gov/view/cdc/169628); only selected variables were reported because of small cell sizes. Sex, race and ethnicity, and other recorded characteristics were documented by coroner or medical examiner reports or law enforcement reports; as such, these might not align with how the victim or suspect self-identifies. This activity was reviewed by CDC, deemed not research, and was conducted consistent with applicable Federal law and CDC policy.*

**TABLE 1:** Number and percentage of intimate partner violence–related homicides* of Hispanic and Latino persons, by victim sex and incident characteristics — National Violent Death Reporting System, 49 states, District of Columbia, and Puerto Rico, 2003–2021

Characteristic	Victim sex		
	Female	Male	Total
	No. (%)	No. (%)	No. (%)
Homicide with known circumstances (% of total)	3,011 (76.2)	14,726 (71.4)	**17,737 (72.2)**
IPV-related homicide	1,453 (48.2)	991 (6.7)	**2,444 (13.8)**
**Total**	**3,954 (16.1)**	**20,627 (83.9)**	**24,581 (100.0)**

**Abbreviation:** IPV = intimate partner violence.

* IPV-related homicides were defined as those involving homicides committed by intimate partners (i.e., victim was an intimate partner or spouse [current, former, or unspecified] of the suspect), corollary victims of intimate partner homicide (i.e., other deaths associated with IPV, including homicides of victims who were not the intimate partner, e.g., children, family, friends, others who intervened in IPV, first responders, and bystanders), or homicides precipitated by jealousy or distress over an intimate partner’s relationship or suspected relationship with another person.

**TABLE 2:** Number and percentage* of intimate partner violence–related homicides^†^ of Hispanic and Latino persons, by victim sex and selected demographic and incident characteristics — National Violent Death Reporting System, 49 states, District of Columbia, and Puerto Rico, 2003–2021

Characteristic	Victim sex		
	Female	Male	Total
	No. (%)	No. (%)	No. (%)
**Victim aged ≥18 yrs**	1,402 (96.5)	939 (94.8)	**2,341 (95.8)**
**Victims of reproductive age with known pregnancy status^§^**	359 (31.4)	NA	**NA**
**Pregnant or ≤1 year postpartum^¶^**	71 (19.8)	NA	**NA**
**Age group, yrs**			
<1	6 (0.4)	7 (0.7)	**13 (0.5)**
1–9	16 (1.1)	16 (1.6)	**32 (1.3)**
10–17	29 (2.0)	29 (2.9)	**58 (2.4)**
18–24	263 (18.1)	234 (23.6)	**497 (20.3)**
25–34	481 (33.1)	342 (34.5)	**823 (33.7)**
35–44	377 (26.0)	224 (22.6)	**601 (24.6)**
45–54	196 (13.5)	88 (8.7)	**284 (11.6)**
55–64	56 (3.9)	38 (3.8)	**94 (3.9)**
>64	29 (2.0)	13 (1.3)	**42 (1.7)**
**Race**			
American Indian or Alaska Native	11 (0.8)	8 (0.8)	**19 (0.8)**
Asian or Pacific Islander	9 (0.6)	5 (0.5)	**14 (0.6)**
Black	64 (4.4)	67 (6.8)	**131 (5.4)**
White	1,150 (79.2)	801 (80.8)	**1,951 (79.8)**
Two or more races	23 (1.6)	15 (1.5)	**38 (1.6)**
Other, unspecified, or unknown	196 (13.5)	95 (9.6)	**291 (11.9)**
**Place of birth**			
United States	836 (57.5)	689 (69.5)	**1,525 (62.4)**
Outside United States	485 (33.4)	244 (24.6)	**729 (29.8)**
Missing or unknown	132 (9.1)	58 (5.9)	**190 (7.8)**
Education**			
Less than high school graduate or GED certificate equivalent	339 (24.2)	293 (31.2)	**632 (27.0)**
High school graduate or GED certificate equivalent	462 (33.0)	342 (36.4)	**804 (34.3)**
Some college or more	366 (26.1)	139 (14.8)	**505 (21.6)**
Unknown	235 (16.8)	165 (17.6)	**400 (17.1)**
**Method of injury**			
Firearm	766 (52.7)	676 (68.2)	**1,442 (59.0)**
Sharp instrument	382 (26.3)	214 (21.6)	**596 (24.4)**
Blunt instrument	64 (4.4)	29 (2.9)	**93 (3.8)**
Personal weapon (e.g., feet and fists)	43 (3.0)	17 (1.7)	**60 (2.5)**
Hanging, strangulation, or suffocation	143 (9.8)	19 (1.9)	**162 (6.6)**
Other method	39 (2.7)	31 (3.1)	**70 (2.9)**
Unknown	16 (1.1)	5 (0.5)	**21 (0.9)**
**Known residence**	1,453 (100.0)	990 (99.9)	**2,443 (100.0)**
**Known injury location**	1,449 (99.7)	987 (99.6)	**2,436 (99.7)**
**Metropolitan status^††^**			
Metropolitan resident	1,260 (86.7)	849 (85.8)	**2,109 (86.3)**
Metropolitan injury location	1,195 (82.5)	814 (82.5)	**2,009 (82.5)**
**Location of injury^††^**			
House or apartment	1,092 (75.2)	542 (54.7)	**1,634 (66.9)**
Street or highway	83 (5.7)	191 (19.3)	**274 (11.2)**
Natural area	35 (2.4)	16 (1.6)	**51 (2.1)**
Motor vehicle	75 (5.2)	80 (8.1)	**155 (6.3)**
Parking lot, public garage, or public transport	51 (3.5)	66 (6.7)	**117 (4.8)**
Other location	85 (5.9)	86 (8.7)	**171 (7.0)**
Unknown	32 (2.2)	10 (1.0)	**42 (1.7)**
Injured at victim’s home	939 (64.8)	337 (34.1)	**1,276 (52.3)**
**Total**	**1,453 (59.5)**	**991 (40.6)**	**2,444 (100.0)**

**Abbreviations:** GED = General Education Development; IPV = intimate partner violence; NA = not applicable.

* Percentages might not sum to 100% because of rounding.

^†^ IPV-related homicides were defined as those involving homicides committed by intimate partners (i.e., victim was an intimate partner or spouse [current, former, or unspecified] of the suspect), corollary victims of intimate partner homicide (i.e., other deaths associated with IPV, including homicides of victims who were not the intimate partner, e.g., children, family, friends, others who intervened in IPV, first responders, and bystanders), or homicides precipitated by jealousy or distress over an intimate partner’s relationship or suspected relationship with another person.

^§^ Proportion among women of reproductive age (15–44 years; n = 1,444).

^¶^ Proportion among women of reproductive age (15–44 years) with known pregnancy status.

** Proportion among victims aged ≥18 years at time of death.

^††^ Proportion among victims with known injury location.

**TABLE 3:** Suspect characteristics for intimate partner violence–related homicides* of Hispanic and Latino persons, by victim sex — National Violent Death Reporting System,^†^ 49 states, District of Columbia, and Puerto Rico, 2003–2021

Characteristic	Victim sex		
	Female	Male	Total
	No. (%)^§^	No. (%)^§^	No. (%)^§^
**Suspect age group, yrs**			
<18	5 (0.3)	36 (3.6)	**41 (1.7)**
18–24	140 (9.6)	177 (17.9)	**317 (13.0)**
25–34	388 (26.7)	249 (25.1)	**637 (26.1)**
35–44	317 (21.8)	142 (14.3)	**459 (18.8)**
45–54	206 (14.2)	59 (6.0)	**265 (10.8)**
55–64	97 (6.7)	24 (2.4)	**121 (5.0)**
>64	258 (17.8)	208 (21.0)	**466 (19.1)**
Unknown	42 (2.9)	96 (9.7)	**138 (5.7)**
**Suspect sex**			
Female	28 (2.0)	206 (22.9)	**234 (10.1)**
Male	1,274 (89.8)	632 (70.1)	**1,906 (82.2)**
Unknown	116 (8.2)	63 (7.0)	**179 (7.7)**
**Suspect ethnicity**			
Hispanic or Latino	728 (51.3)	434 (48.2)	**1,162 (50.1)**
Unknown	430 (30.3)	317 (35.2)	**747 (32.2)**
Suspect race and ethnicity^¶,^**			
American Indian or Alaska Native	7 (0.5)	6 (0.7)	**13 (0.6)**
Asian or Pacific Islander	10 (0.7)	8 (0.9)	**18 (0.8)**
Black	156 (11.0)	90 (10.0)	**246 (10.6)**
Hispanic or Latino	26 (16.7)	36 (40.0)	**62 (25.2)**
Not Hispanic or Latino or unknown	130 (83.3)	54 (60.0)	**184 (74.8)**
White	690 (48.7)	440 (48.8)	**1,130 (48.7)**
Hispanic or Latino	509 (73.8)	298 (67.7)	**807 (71.4)**
Not Hispanic or Latino or unknown	181 (26.2)	142 (32.3)	**323 (28.6)**
Two or more races	17 (1.2)	7 (0.8)	**24 (1.0)**
Hispanic or Latino	13 (76.5)	6 (85.7)	**19 (79.2)**
Not Hispanic or Latino or unknown	4 (23.5)	1 (14.3)	**5 (20.8)**
Other, unspecified, or unknown	538 (37.9)	350 (38.9)	**888 (38.3)**
Hispanic or Latino	180 (33.5)	93 (26.6)	**273 (30.7)**
Not Hispanic or Latino or unknown	358 (66.5)	257 (73.4)	**615 (69.3)**
**Victim’s relationship to suspect**			
Intimate partner	1,205 (85.0)	236 (26.2)	**1,441 (62.1)**
Current intimate partner	907 (75.3)	188 (79.7)	**1,095 (76.0)**
Former intimate partner	256 (21.2)	40 (17.0)	**296 (20.5)**
Intimate partner, or unknown whether current or former intimate partner	42 (3.5)	8 (3.4)	**50 (3.5)**
Nonintimate partner (i.e., corollary victim)	87 (6.1)	529 (58.7)	**616 (26.6)**
Acquaintance or friend	11 (0.8)	129 (14.3)	**140 (6.0)**
Child	28 (2.0)	44 (4.9)	**72 (3.1)**
Parent	2 (0.1)	12 (1.3)	**14 (0.6)**
Other relative	9 (0.6)	17 (1.9)	**26 (1.1)**
Romantic jealousy	1 (0.1)	64 (7.1)	**65 (2.8)**
Other known person	32 (2.3)	198 (22.0)	**230 (9.9)**
Other (e.g., rival gang member, law enforcement, or stranger)	4 (0.3)	65 (7.2)	**69 (3.0)**
Unknown	126 (8.9)	136 (15.1)	**262 (11.3)**
**IPV-related homicides with known suspects**	1,418 (97.6)	901 (90.9)	**2,319 (94.9)**
**Total**	**1,453 (59.5)**	**991 (40.6)**	**2,444 (100.0)**

**Abbreviation:** IPV = intimate partner violence.

* IPV-related deaths were defined as those involving homicides committed by intimate partners (i.e., victim was an intimate partner or spouse [current, former, or unspecified] of the suspect); corollary victims of intimate partner homicide (i.e., other deaths associated with IPV, including homicides of victims who were not the intimate partner, e.g., family, friends, others who intervened in IPV, first responders, and bystanders); or homicides precipitated by jealousy or distress over an intimate partner’s relationship or suspected relationship with another person.

^†^ All proportions based on the number of homicides with known suspects.

^§^ Percentages might not sum to 100% because of rounding.

^¶^ Hispanic and Latino persons can be of any race.

** Stratification by ethnicity is only shown when the sample size is sufficient (>5 per cell).

**TABLE 4:** Circumstances of intimate partner violence–related homicides* of Hispanic and Latino persons, by victim sex — National Violent Death Reporting System,^†^ 49 states, District of Columbia, and Puerto Rico, 2003–2021

Circumstance	Victim sex		
	Female	Male	Total
	No. (%)^§^	No. (%)^§^	No. (%)^§^
IPV-related homicides with known circumstances	1,445 (99.5)	987 (99.6)	**2,432 (99.5)**
IPV-related homicides with known suspects	1,418 (97.6)	901 (90.9)	**2,319 (94.9)**
**Interpersonal**			
IPV-related	1,426 (98.7)	858 (86.9)	**2,284 (93.9)**
Family relationship problem^¶^	32 (2.6)	27 (3.3)	**59 (2.9)**
Other relationship problem (nonintimate)	25 (1.7)	92 (9.3)	**117 (4.8)**
Jealousy	187 (12.9)	494 (50.1)	**681 (28.0)**
Victim of IPV during past month	120 (8.3)	16 (1.6)	**136 (5.6)**
Perpetrator of IPV during past month	11 (0.8)	60 (6.1)	**71 (2.9)**
**Life stressor**			
Argument or conflict	628 (43.5)	459 (46.5)	**1,087 (44.7)**
Physical fight (two persons, not a brawl)**	114 (10.6)	176 (24.1)	**290 (16.0)**
Crisis during previous or upcoming 2 weeks	138 (9.6)	132 (13.4)	**270 (11.1)**
History of child abuse or neglect	4 (0.3)	2 (0.2)	**6 (0.3)**
**Crime and criminal activity**			
Precipitated by another crime	137 (9.5)	180 (18.2)	**317 (13.0)**
Crime in progress^††^	65 (47.5)	106 (58.9)	**171 (53.9)**
Drug involvement	27 (1.9)	45 (4.6)	**72 (3.0)**
Gang related	22 (1.5)	44 (4.5)	**66 (2.7)**
**Homicide event**			
Victim used a weapon	15 (1.0)	79 (8.0)	**94 (3.9)**
Caretaker abuse or neglect led to death**	37 (3.4)	11 (1.5)	**48 (2.7)**
Brawl	2 (0.1)	26 (2.6)	**28 (1.2)**
Random violence^¶^	7 (0.6)	6 (0.7)	**13 (0.6)**
Justifiable self-defense	2 (0.1)	47 (4.8)	**49 (2.0)**
Drive-by shooting^¶^	8 (0.6)	60 (6.1)	**68 (2.8)**
Mentally ill suspect^§§^	55 (3.9)	16 (1.8)	**71 (3.1)**
Walk-by assault**	10 (0.9)	16 (2.2)	**26 (1.4)**
Victim was a bystander	4 (0.3)	23 (2.5)	**29 (1.2)**
Victim was an intervener assisting a crime victim	5 (0.4)	15 (1.5)	**20 (0.8)**
Stalking**	33 (3.1)	16 (2.2)	**49 (2.7)**
**Total**	**1,453 (59.5)**	**991 (40.6)**	**2,444 (100.0)**

**Abbreviation:** IPV = intimate partner violence.

* IPV-related deaths were defined as those involving homicides committed by intimate partners (i.e., victim was an intimate partner or spouse [current, former, or unspecified] of the suspect), corollary victims of intimate partner homicide (i.e., other deaths associated with IPV, including homicides of victims who were not the intimate partner, e.g., family, friends, others who intervened in IPV, first responders, and bystanders), or homicides precipitated by jealousy or distress over an intimate partner’s relationship or suspected relationship with another person.

^†^ Proportions are based on the total number of homicides with known circumstances, unless otherwise noted. More than one circumstance can be reported.

^§^ Percentages might not sum to 100% because of rounding.

^¶^ Variable collected since 2009. Proportions are based on homicides with known circumstances during 2009–2021 (male = 825; female = 1,222; total = 2,047).

** Variable collected since 2013. Proportions are based on homicides with known circumstances during 2013–2020 (male = 731; female = 1,078; total = 1,809).

^††^ Proportion based on the number of homicides precipitated by another crime.

^§§^ Proportion among those homicides with known suspects.

## Results

### Characteristics of Hispanic and Latino IPV-Related Homicide Victims

During 2003–2021, a total of 24,581 homicides of Hispanic and Latino persons were reported in NVDRS, and 17,737 (72.2%) had known circumstances. Of the homicides among Hispanic and Latino persons with known circumstances, 13.8% (2,444 of 17,737) were determined to be IPV-related (Table 1). Of these IPV-related homicides, 59.5% (n = 1,453) were female victims (Table 2); conversely, female victims comprised the minority of total homicide victims (n = 3,954; 16.1%) (Supplementary Table 2, https://stacks.cdc.gov/view/cdc/169628). In Puerto Rico, 8.5% (254 of 2,986) of homicides with known circumstances were IPV-related; 32.7% (n = 83) of IPV-related homicide victims were female (Supplementary Table 5, https://stacks.cdc.gov/view/cdc/169628).

Among Hispanic and Latino IPV-related homicide victims (n = 2,444), approximately eight of 10 were identified as White (n = 1,951; 79.8%) and 5.4% (n = 131) as Black. In Puerto Rico, 208 of 255 (81.9%) of IPV-related homicide victims were identified as White and 46 (18.1%) as Black. In the national dataset of Hispanic and Latino IPV-related homicide victims, one third of females (n = 485; 33.4%) and one fourth of males (n = 244; 24.6%) were born outside of the United States. Approximately one third (34.3%) of victims had completed high school or earned a General Education Development certificate (female: n = 462; 33.0%; male: n = 342; 36.4%) and 1 in 4 (n = 366; 26.1%) female victims and 14.8% (n = 139) of male victims had completed some college.

Among female Hispanic and Latino victims of IPV-related homicides, 78.7% (n = 1,144) were of childbearing age (15–44 years) and 31.4% (n = 359) had a known pregnancy status recorded. Of those with known pregnancy status, 19.8% (n = 71) were pregnant or ≤1 year postpartum at the time of death. This result was similar for all Hispanic and Latino female homicide victims (i.e., both IPV-related and non–IPV-related homicide victims; n = 3,954); 66.8% (n = 2,640) victims were of childbearing age (15–44 years) and 31.4% (n = 830) had their pregnancy status recorded (Supplementary Table 2, https://stacks.cdc.gov/view/cdc/169628). Of those with a known pregnancy status, 18.6% (n = 154) were pregnant or ≤1 year postpartum at the time of death.

A firearm was used in 59% of the 2,444 Hispanic and Latino IPV-related homicides (female: n = 766; 52.7%; male: n = 676; 68.2%); a firearm also was used in 200 of the 254 IPV-related homicides in Puerto Rico (male: n = 148; 86.6%; female: n = 52; 62.7%). In the national dataset of Hispanic and Latino IPV-related homicides (n = 2,444), approximately one tenth (n = 143; 9.8%) of female and 1.9% (n = 19) of male victims died by hanging or strangulation. Approximately two thirds (n = 939; 64.8%) of IPV-related homicides of female victims and one third (n = 337; 34.1%) of male victims occurred in the victim’s home. This result differed from total Hispanic and Latino homicides (n = 24,581; Supplementary Table 2, https://stacks.cdc.gov/view/cdc/169628) in which approximately half of female homicides (n = 1,809; 51.7%) and nearly one in five male homicides (n = 3,485; 19.4%) occurred in the victim’s own home.

### Characteristics of Hispanic and Latino IPV-Related Homicide Suspects

Of the 2,444 Hispanic and Latino IPV-related victims in NVDRS with known circumstances, 94.9% (n = 2,319) had known suspects (Table 3). Most suspects were male (n = 1,906; 82.2%), regardless of the sex of the victim. Males were identified as suspects for 89.8% (n = 1,274) of female victims, and 70.1% (n = 632) of male victims. Half of suspects (n = 1,162; 50.1%) were identified as Hispanic or Latino persons, 13.9% (323 of 2,319) of suspects were non-Hispanic White, and 7.9% (184 of 2,319) were non-Hispanic Black.

The victim-suspect relationship for Hispanic and Latino IPV-related homicides with known suspects (n = 2,319) differed by sex. For female victims, 1,205 of 1,418 (85.0%) of suspects were current or former intimate partners. Of these intimate partners, three fourths were current intimate partners (n = 907; 75.3%). In Puerto Rico, 75 of 85 (93.8%) suspects of female IPV-related homicide victims were current or former partners. In contrast, 236 of 901 (26.2%) suspects of male Hispanic and Latino IPV-related homicide victims in the national dataset were current or former intimate partners; in Puerto Rico, this percentage was lower (n = 15 of 116; 12.9%). Most male victims of IPV-related homicides in the national dataset were corollary victims; 529 of 901 (58.7%) suspects were known to the victim but were not current or former partners. Examples of other relationships to suspects include friends or acquaintances (n = 129; 14.3%) or another person known to the victim (n = 198; 22.0%; e.g., a bystander witnessed a person assaulting their spouse and when the bystander tried to intervene, they were shot by the perpetrator). Seventy-two children were identified as corollary victims; 44 were male and 28 were female.

### Circumstances of IPV-Related Homicides of Hispanic and Latino Persons

Of the 2,432 IPV-related homicides of Hispanic and Latino victims with known circumstances, IPV was specifically mentioned in the medical examiner or law enforcement narrative for 98.7% (n = 1,426) of female victims and 86.9% (n = 858) of male victims (Table 4). Jealousy was mentioned for approximately half (n = 494; 50.1%) of male victims and 12.9% (n = 187) of female victims. During the month before the fatal injury, 8.3% (n = 120) of females and 1.6% (n = 16) of males also were known to be victims of interpersonal violence (of any kind). The perpetration of interpersonal violence (of any kind) during the month before fatal injury was noted for 6.1% (n = 60) of male victims and 0.8% (n = 11) of female victims.

More than two fifths (n = 1,087; 44.7%) of all IPV-related homicides of Hispanic and Latino persons were precipitated by an argument or conflict. A physical fight preceded nearly one fourth (n = 176; 24.1%) of male IPV-related homicides and 10.6% (n = 114) of female IPV-related homicides. Having a crisis (e.g., eviction notice or upcoming court date) during the previous or upcoming 2 weeks around the IPV-related homicide was noted for 13.4% (n = 132) of male victims and 9.6% (n = 138) of female victims. Approximately one in five male (n = 180; 18.2%) and one in 10 female IPV-related homicides (n = 137; 9.5%) were precipitated by another crime. A small proportion of IPV-related homicides was precipitated by drug involvement (male: n = 45; 4.6%; female: n = 27; 1.9%) or gang-related activity (male: n = 44; 4.5%; female: n = 22; 1.5%). Weapons use by the victim and drive-by shootings were reported for 8.0% (n = 79) and 6.1% (n = 60) of male victims, respectively, and 1.0% (n = 15) and 0.6% (n = 8) of female victims, respectively.

## Discussion

This report provides the first focused, descriptive overview of IPV-related homicides of Hispanic and Latino persons in the United States using NVDRS, a state-based surveillance system that includes circumstance information from multiple sources. Findings highlight differences in IPV-related homicides among Hispanic and Latino persons by sex. Approximately half of Hispanic and Latino female homicides were IPV-related; 6.7% of Hispanic and Latino male homicides were IPV-related. Similar findings have been reported in previous analyses of NVDRS data on IPV-related homicides among other races and ethnicities (*5*,*6*,*43*). These findings have important implications for preventing the escalation of IPV to IPH and for the primary prevention of IPV (*46*).

### Preventing the Escalation of IPV to IPH

Most suspects in Hispanic and Latino IPV-related homicides of female victims were current or former intimate partners (85.0%) and more than two fifths (44.7%) of all IPV-related homicides were precipitated by an argument or conflict. Preventing the escalation of IPV to IPH requires creating accessible and high-quality avenues of support for IPV victims including safety planning, establishing financial independence, safe and stable housing, child care, emotional support, and legal advocacy (*47*). Although this report did not examine victims’ previous interactions with law enforcement, previous research has documented that IPV survivors, in general, can face substantial barriers to accessing police and community support (*48*). These barriers are further exacerbated for certain Hispanic and Latino IPV survivors because they might not feel comfortable calling the police for fear of experiencing racism, discrimination (e.g., because of immigration status), disbelief, victimization by police themselves, or fear of children being removed from the home (*21*,*47*,*49*). Research in Oklahoma found that strong community-level relationships with police can improve access to care, and strong protocols for first responders can increase support for IPV victims (*50*,*51*). Studies have documented multiple promising risk-informed collaborations between police and community service partners to increase victim support and reduce subsequent violence (*50*–*52*).

Certain Hispanic and Latino IPV survivors and victims who are immigrants could face additional fears when seeking help, including fears of deportation (*21*). Research has documented that perpetrators of IPV sometimes use visa status or misinformation about immigration policy to prevent IPV victims from accessing help or calling police (*21*). At the community level, previous research has found that calls to law enforcement regarding IPV decrease when immigration enforcement in the community increases (*53*). Furthermore, another study found that living in a community with sanctuary policies (i.e., policies that regulate cooperation between local authorities and Federal immigration authorities) was associated with lower IPH among female Hispanic and Latino persons (*54*).

Firearms were the most common mechanism of IPV-related homicides of Hispanic and Latino persons. This finding aligns with IPH in the United States overall, where half of IPHs are committed using firearms (*55*). From 2019 to 2020, the firearm homicide rate increased by approximately 35% (*56*,*57*); the firearm homicide rate for all races and ethnicities in 2021 was the highest recorded in more than two decades and then declined slightly in 2022 (*57*). Substantial inequities in firearm homicide by race and ethnicity exist (*57*). Research has found promising strategies to reduce the risk for lethal violence. For example, a recent review described moderate evidence that state laws prohibiting firearm access among those subject to domestic violence restraining orders are associated with a decrease in total and firearm-related IPHs (*58*).

Another key finding from this report is that 19.8% of all IPV-related homicides of female victims with known pregnancy status were either pregnant or ≤1 year postpartum at the time of death. This result is consistent with previous NVDRS analyses that identified a significant positive association between a history of IPV and pregnancy-associated homicides (*5*,*6*,*59*). The relation between pregnancy and IPV might result from reproductive coercion, a continuation of prepregnancy IPV, partners using pregnancy as a controlling mechanism, or partner stress that is due to feeling out of control because of the pregnancy (*60*). Paid parental leave has been found to be protective against IPV, as well as child abuse (*61*,*62*); however, research has found that Hispanic and Latino parents are significantly less likely to have access to paid parental leave compared with their non-Hispanic White counterparts (*63*). Few states mandate paid parental leave, and Hispanic and Latino women are overrepresented in sectors and positions that do not offer such benefits (*64*). Additional research is needed to understand if and how paid parental leave could be protective against IPH of Hispanic and Latino persons. Home-visiting programs; patient-centered prenatal, postpartum, maternal, and child health care; and universal screening from health care providers for IPV, substance use, and mental health problems to connect patients with resources also can help address IPV and promote maternal health (*46*,*62*,*65*,*66*).

### Primary Prevention of IPV

CDC has identified multiple evidence-based strategies to prevent IPV before it occurs, including teaching safe and healthy relationship skills, engaging influential adults and peers, disrupting the developmental pathways toward IPV, creating protective environments, and strengthening economic supports for families (*62*). Although these strategies have broad prevention implications for many different communities, few IPV prevention strategies have been specifically tailored to and evaluated for Hispanic and Latino persons (*67*). Furthermore, Hispanic and Latino communities are heterogeneous groups, and consideration should be made for the variation in IPV risk and protective factors across Hispanic and Latino subgroups (*68*–*70*).

This report found that 29.8% of all Hispanic and Latino IPV-related homicide victims were born outside the United States, similar to national estimates (*71*). Although citizenship and documentation status were not explored in this report, these can be important determinants for access to factors known to be protective from IPV and IPH. Documentation status (e.g., refugee status and having a work visa) can influence access to safe employment that provides living wages, health care systems, eligibility for social safety programs, and civic participation (*72*). Whereas the majority of Hispanic and Latino persons in the United States have U.S. citizenship, those who do not have U.S. citizenship might not benefit from multiple policy-level strategies to prevent violence (*19*,*62*,*71*,*73*,*74*). For example, those without documentation are excluded from the health insurance exchange, which limits their access to clinicians who can be an important gateway to help those affected by violence in the home (*75*). Furthermore, earned income tax credits, increased minimum wage, child tax credits, and other social safety net programs that have been documented in reducing child abuse and neglect, and theorized to prevent IPV by reducing household stress, might not be accessible (*62*,*73*).

## Limitations

The findings in this report are subject to at least six limitations. First, although this report analyzes data from 49 states, Puerto Rico, and the District of Columbia, the data are not nationally representative because not all states and territories participate or participate fully. Furthermore, certain jurisdictions (e.g., Puerto Rico) have included data in NVDRS for a shorter amount of time. As such, the number of homicides accounted for in NVDRS is underreported compared with the actual number of homicides in the United States. Second, the prevalence of IPV-related homicides could be underestimated because the perpetrator of the homicide or their relationship to the victim might be unknown (*76*). Third, abstractors are limited to information in source documents, which is reflected in the high percentage of missing data regarding pregnancy among female victims of reproductive age. Pregnancy status might be more likely to be available for those who are or were recently pregnant and missing for those who were not pregnant, thus inflating the percentage of IPH victims who were pregnant. However, pregnant women also could have been excluded from both the numerator and denominator, especially those early in their pregnancy or if a recent pregnancy did not result in a live birth (e.g., miscarried or still births). The findings for pregnancy status should be interpreted with caution. Fourth, recorded information might not align with victim or suspect’s self-identification (e.g., race and ethnicity and gender). Hispanic and Latino victims who were ethnically misclassified by law enforcement and medical examiners might have been excluded from this dataset. Alternatively, persons who do not identify as Hispanic and Latino could have been misclassified and included in the dataset (*3*). Fifth, data on IPH that has occurred on tribal lands might be held in tribal and Federal data systems and therefore not included in this dataset (*6*). Finally, these findings do not examine sexual orientation, gender identity, or gender expression among homicide victims, although previous research has documented high IPV victimization among persons who identify as a sexual or gender minority (*77*,*78*). Previous studies of death certificates have documented that these indicators tend to be underreported and misclassified (*45*,*79*). Increasing capacity and consistency of those completing source documents to document these characteristics is critical and would help address the substantial gap in research on IPV-related homicides among those who identify as a sexual or gender minority (*50*,*80*). Future research efforts can endeavor to better understand the epidemiological profile of IPV-related homicides of persons who hold both Hispanic or Latino and other intersectional identities.

## Future Directions

Future studies and prevention efforts can incorporate research findings as well as build on Hispanic and Latino communities’ longstanding traditions of countering adversities. For example, community-led efforts have deconstructed some structural barriers, including addressing racism and nativism in schools, increasing workplace protections, creating fair and affordable financial products (e.g., opportunities to build credit and access to wealth-building loans), and expanding pathways to citizenship for those born outside the United States (*81*–*84*). Decades of transnational feminist work also have sought to end violence against Latino women (*85*). Similarly, AI/AN communities are leading efforts to stop the erasure of AI/AN homicide victims, specifically by upholding the pillars of wellness, protection, self-determination, and resilience found in Traditional Indigenous Knowledge, cultural knowledge, spiritual and ancient healing, and health systems (*36*,*86*,*87*). These efforts could benefit Hispanic and Latino persons who share AI/AN ancestry and could inform future prevention research.

The findings in this report can be used by Federal, tribal, state, and local governments; public health and health services sectors; the criminal justice system; and victim services to inform primary and secondary IPV prevention efforts. Future research can evaluate policies that create more inclusive social safety net programs that prevent further economic and social marginalization and decrease risk for experiencing violence (*61*,*88*). For example, affordable housing through the Low-Income Housing Tax Credit program, at the state level, is associated with a decrease in homicides in the context of IPV (*89*). Similarly, early childhood education programs (e.g., Head Start), have been documented to prevent multiple forms of violence (*61*). Fully implementing the Culturally and Linguistically Appropriate Service standards (*90*) and extending to other sectors such as police departments (*91*) could increase opportunities for intervention before IPV escalates to homicide. Providing trauma-informed care, especially within a collaborative network approach, is another secondary prevention avenue (*88*,*92*,*93*).

Future research also can examine how multiple cultural and racial identities can place subgroups within the Hispanic and Latino population (e.g., AI/AN and Black Hispanic and Latino persons) at increased risk for violence and health inequities (*94*,*95*). Although the study data are not representative of the entire Hispanic and Latino population in the United States and racial and ethnic misclassification could be possible, the percentage of IPV-related homicide victims identified as Black was slightly higher than national estimates (*12*). Examining the links between IPH and the effects of anti-Black sentiment, the denial of African and indigenous ancestry, and the mistreatment of darker-skinned Hispanic and Latino persons (*96*,*97*) could inform IPV and IPH prevention strategies. These strategies could address prejudice and discrimination between persons (personally mediated racism) as well as those that address differential access to the goods, services, and opportunities of society by race (institutional racism) (*98*). Future studies also can evaluate which violence prevention programs and policies are best able to account for the heterogeneity of the Hispanic and Latino population in the United States (*99*,*100*).

## Conclusion

This report on Hispanic and Latino victims of IPV-related homicides found heterogeneity of characteristics and circumstances, particularly in the context of victim sex. Female Hispanic and Latino homicide victims were more likely to be IPH victims compared with males. Firearm injuries accounted for the majority of IPV-related homicides. The findings also raise important questions about the role of pregnancy and intersecting racial identities. Comprehensive efforts to prevent IPH and IPV-related homicides among Hispanic and Latino persons might benefit from consideration of the structural factors that increase risk, community engagement and community-developed solutions, and use of the best available evidence in a way that is culturally and ethnically relevant.

## Footnotes

* 45 C.F.R. part 46, 21 C.F.R. part 56; 42 U.S.C. Sect. 241(d); 5 U.S.C. Sect. 552a; 44 U.S.C. Sect. 3501 et seq.
